# CD24-Fc resolves inflammation and enhances anti-HIV CD8 T cells with polyfunctionality during HIV-1 infection under cART

**DOI:** 10.1371/journal.ppat.1012826

**Published:** 2025-08-08

**Authors:** Guangming Li, Jianping Ma, Haisheng Yu, Ourania Tsahouridis, Yaoxian Lou, Xiuting He, Masaya Funaki, Pan Zheng, Yang Liu, Lishan Su

**Affiliations:** 1 Institute of Human Virology, Departments of Pharmacology, Microbiology and Immunology, University of Maryland School of Medicine, Baltimore, Maryland, United States of America; 2 Lineberger Comprehensive Cancer Center, Department of Microbiology and Immunology, University of North Carolina at Chapel Hill, School of Medicine, Chapel Hill, North Carolina, United States of America; 3 OncoC4, Inc., Rockville, Maryland, United States of America; NIH, NIAID, UNITED STATES OF AMERICA

## Abstract

The persistence of HIV-1 reservoirs during combination anti-retroviral therapy (cART) is associated with chronic inflammation and systemic immune activation in people infected with HIV-1 (PWH), leading to a suboptimal immune reconstitution as well as an increased risk of non-AIDS events. In this study, we assessed the effect of CD24-Fc, a fusion protein with anti-inflammatory properties that interacts with danger-associated molecular patterns (DAMPs) and siglec-10, in humanized mice with chronic HIV-1 infection under suppressive cART in vivo and in peripheral blood mononuclear cells (PBMCs) from PWH in vitro. We report that CD24-Fc treatment significantly reduced inflammation and immune hyperactivation in humanized mice with HIV-1 infection and cART. CD24-Fc treatment improved recovery of CD4 T cells, reduced immune hyper-activation, increased functional central memory T cells. Notably, CD24-Fc treatment increased CXCR5 + CD8 central memory T cells (T_CM_) with increased HIV-specific polyfunctionality in humanized mice and in PBMC from PWH. This enhanced anti-HIV T cell activity was associated with improved control of HIV-1 viral rebound and reduced HIV-1 pathogenesis upon cART cessation. Our findings indicate that CD24-Fc may provide a promising new therapeutic for treating chronic inflammation and associated diseases in PWH.

## Author summary

Current combination antiretroviral therapy (cART) effectively suppresses HIV-1 infection but fails to cure HIV-1 in people with HIV (PWH). Persistence of HIV-1 reservoir cells contribute to chronic immune activation and inflammation associated diseases. We tested the therapeutic effect of the anti-inflammatory fusion protein CD24-Fc in humanized mice with HIV-1 infection and suppressive cART in vivo and in PMBCs from PWH with cART in vitro. We report here that CD24-Fc resolved chronic inflammation and HIV-1 immune pathogenesis, enhanced CD8 memory T cells with antiviral functions in humanized mice with HIV and cART, and in PWH PBMCs. CD24-Fc delayed virus rebound and reduced viral pathogenesis after cART cessation. CD24-Fc could provide a novel therapeutic strategy for treating HIV-associated chronic systemic inflammation and its associated diseases in PWH.

## Introduction

The persistence of the HIV-1 reservoir in people infected with HIV (PWH) during combination antiretroviral therapy (cART) prevents HIV cure [1,2]. It requires lifelong cART adherence due to the presence of HIV-1 reservoirs, which cause rapid viral rebound if treatment is interrupted. HIV-1 reservoir persistence is often accompanied by residual inflammation that impairs anti-HIV immunity and promotes systemic inflammatory diseases [3]. Over 30% of PWH who initiate cART with low CD4 counts experience poor CD4 T cell reconstitution, correlated with a higher risk of non-AIDS-related complications [4–8]. Furthermore, individuals with suboptimal CD4 recovery tend to exhibit greater immune activation and inflammation compared to those with better CD4 recovery [9], while PLWH who continue to experience inflammation despite effective cART are at an elevated risk for comorbidities and non-AIDS events [10–12]. Therefore, persistent inflammation and immune hyper-activation play critical roles in HIV-1 associated diseases in post cART era. Targeting residual inflammation in PWH under cART may offer a promising therapeutic avenue for managing HIV-1 and related diseases.

HIV-1 infection induces cell death both directly and indirectly through various pathways [13–17], resulting in cell death, immune activation, inflammation, and tissue damage even during effective cART [9,18–21]. Inflammatory responses triggered by cell death and tissue damage are well-documented in chronic diseases [22,23], where danger-associated molecular patterns (DAMPs) released during cellular stress promote inflammation and immune activation [24,25], play a crucial role in promoting inflammatory response and immune activation during cell death or tissue damage [26]. Blocking DAMP signaling may thus offer a new anti-inflammatory approach for managing chronic HIV-1 disease.

Recent studies have explored therapeutic modulation of the inflammatory response in PWH, including anti-inflammatory drugs targeting specific receptors or cytokines, as well as immunomodulatory supplements [27]. Although some treatments reduced immune activation and inflammation associated with HIV-1, none significantly improved anti-HIV immune responses or reservoir elimination. In humanized mouse models, we and others have demonstrated that blocking type I interferon (IFN-I) signaling during cART reduces systemic inflammation and immune activation, enhancing anti-HIV immunity and promoting HIV-1 reservoir reduction [28,29]. We have also reported that blocking IFN-I receptors or depleting IFN-I-producing cells in HIV-infected mice increased human immune cell number and function [30,31]. These findings underscore the potential of anti-inflammatory therapies as novel immunotherapies for HIV-1 associated diseases.

In non-human primates (NHPs) infected with SIV, the human CD24-Fc fusion protein, which mitigates inflammation through interactions with DAMPs and Siglec-10 on myeloid cells. CD24-Fc conferred protection against SIV-induced wasting syndrome, intractable diarrhea, and decreased AIDS morbidity and mortality in NHP with pathogenic SIV infection [32,33]. In a phase II clinical trial with graft-versus-host disease (GVHD) patients, CD24-Fc was well tolerated and led to reduced severe acute GVHD and high survival rates [34]. Additionally, a recent phase III trial (NCT04317040) demonstrated that CD24-Fc effectively reduced systemic inflammation and promoted immune homeostasis in severe COVID-19 patients, without compromising the anti-viral antibody response [35].

We investigated the effects of CD24-Fc on HIV-1 reservoir, persistent inflammation and immune pathogenesis during cART in humanized mice engrafted with human immune cells that support HIV-1 infection and respond to cART with relevant inflammatory pathogenesis [29–31,36–44]. In addition, we confirmed and extended key findings from humanized mice with PBMCs from PWH.

## Results

### CD24-Fc treatment resolves the residual inflammation during chronic HIV-1 infection under suppressive cART

**Fig 1:**
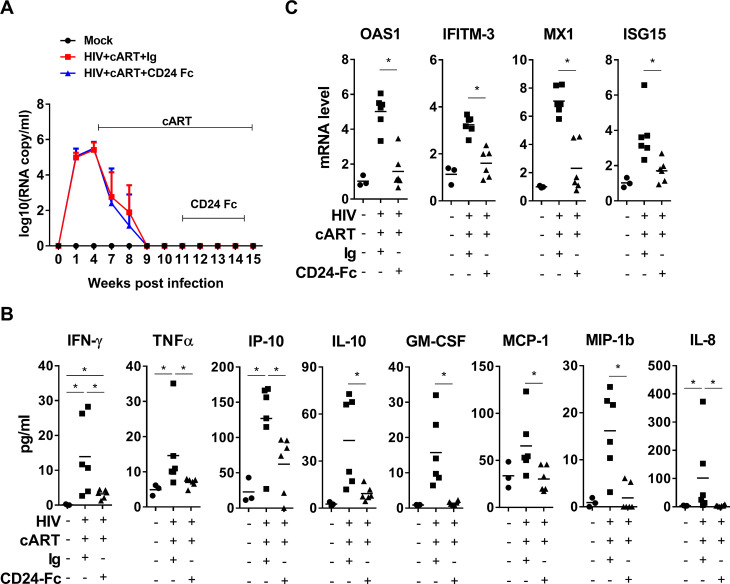
CD24-Fc resolves inflammation during HIV infection with cART. Humanized mice were infected, treated and terminated at 15 wpi. **(A)** Plasma HIV-1 viral load during experiment. Error bars in Fig 1A indicate mean values ± s.e.m. **(B)** Cytokines in blood measured by Luminex. (C) mRNA level of ISGs in splenocytes detect by real-time PCR. Bars represent mean values. *P* values calculated using the ordinary one-way ANOVA Turkey’s multiple comparisons test. * = *p* < 0.05.

To study the effect of CD24-Fc on persistent HIV-1 diseases under combination antiretroviral therapy (cART), we used the well-established humanized mouse model infected with the CCR5-tropic HIV-1 JRCSF isolate [45]. The cART was initiated at 4 weeks post-infection (wpi), and HIV-1 viremia was suppressed to undetectable levels following 5 weeks of treatment. Starting at 11 wpi (7 weeks with cART), CD24-Fc was injected via intraperitoneal (i.p.) twice weekly, and continued until 3 days before euthanasia at 15 wpi. Notably, no viremia blip was observed in any infected animals following suppression by cART, indicating effective viral inhibition (Fig 1A). At termination, we measured inflammatory cytokines in blood, including IFN-γ, TNF-α,IP-10, IL-10, GM-CSF, MCP-1, MIP-1b and IL-8. CD24-Fc treatment decreased the residual inflammatory cytokines as compared to the cART + IgG control mice (Fig 1B, S1 Table). This reduction in inflammation was further confirmed by significantly decreased levels of interferon-stimulated genes (ISGs) in splenocytes in the CD24-Fc group compared to the cART + IgG group (Fig 1C). Remarkably, plasma inflammatory cytokines and tissue ISG mRNA levels in CD24-Fc treated animals were reduced to those in uninfected animals, indicating that CD24-Fc effectively resolved HIV-associated inflammation that was only partially reduced by suppressive cART (Fig 1B and 1C).

### CD24-Fc treatment increases T cell recovery including CD8 central memory T cells in vivo

**Fig 2:**
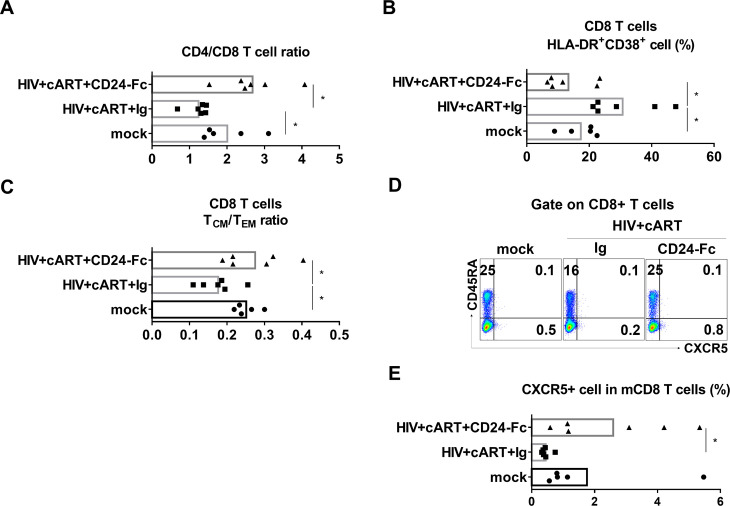
CD24-Fc improves human T cell recovery during HIV infection with cART. Splenocytes were analyzed at termination. **(A)** The ratio of CD4 + /CD8 + T cells. **(B)** The frequency of HLA-DR + CD38 + CD8 + T cells. **(C)** The ratio of central memory/effector memory CD8 + T cells. **(D)** Representative FACS plots show CXCR5 and CD45RA expression in CD8 + T cells. **(E)** Summary graphs show the frequency of CXCR5 + cell in memory CD8 T cells. Bars represent mean values. *P* values calculated using the ordinary one-way ANOVA Turkey’s multiple comparisons test. * = *p* < 0.05.

HIV-1 infection targets CD4 + T cells and leads to their depletion via both direct and indirect mechanisms [46]. Effective cART was able to recover CD4 + T cells in HIV patients. However, incomplete reconstitution of CD4 + T cells occurs in over 30% patients [47,48]. In humanized mice with HIV infection, the cART + IgG control group still showed a significantly lower CD4 + /CD8 + T cell ratio in the spleen compared to the uninfected (mock) group. In contrast, CD24-Fc treatment further increased the CD4 + /CD8 + T cell ratio relative to the cART + IgG group (Figs 2A and S1A). However, there was no significant difference in the total number of human immune cells among groups (S1B Fig).

Although cART effectively suppressed viral replication, infected animals displayed persistent immune hyper-activation, evidenced by an elevated frequency of HLA-DR and CD38 double-positive CD8 + T cells in the cART + IgG group (Figs 2B and S1C) and as reported [29]. CD24-Fc treatment significantly reduced immune activation in comparison with both the cART + IgG and mock control groups, indicating effective resolution of immune activation (Figs 2B and S1C). Chronic HIV-1 infection results in the depletion of central memory CD8 + T (T_CM_) cells, which do not fully recover despite effective viral suppression in PWH [49]. Similarly, we observed that HIV-1 infection in humanized mice led to reduced T_CM_ cell frequency and a lower T_CM_/effector memory T cell (T_EM_) ratio, even with cART-mediated viral suppression (Figs 2C and S1D). Remarkably, CD24-Fc treatment in combination with cART increased T_CM_ cell levels and the T_CM_/T_EM_ ratio to that of uninfected animals (Figs 2C and S1D). We also measured PD-1 expression and found the elevated PD-1 expression in HIV/cART-treated mice was not reduced by CD24-Fc in either central or effector memory CD8 + T cells (S1E Fig). These findings suggest that HIV-1-induced immune pathogenesis is not resolved by suppressive cART alone. CD24-Fc demonstrated an anti-inflammatory effect, reducing HIV-1-associated chronic immune pathology during cART.

It has been recently reported that CD8 + T cells expressing the chemokine receptor CXCR5 are crucial for controlling chronic viral replication, with its levels inversely correlated with HIV-1 viral load in PWH [50]. We were interested to know whether CD24-Fc treatment affected CXCR5 + CD8 + T cells. We investigated splenocytes and observed an increase of CXCR5 expression specifically in CD8 memory cells in CD24-Fc treatment group compared to IgG group (Fig 2D and 2E). No significant difference in CXCR5 expression in total CD8 + T cells was detected (S1F Fig).

### CD24-Fc treatment increases anti-HIV T cell function in vivo

**Fig 3:**
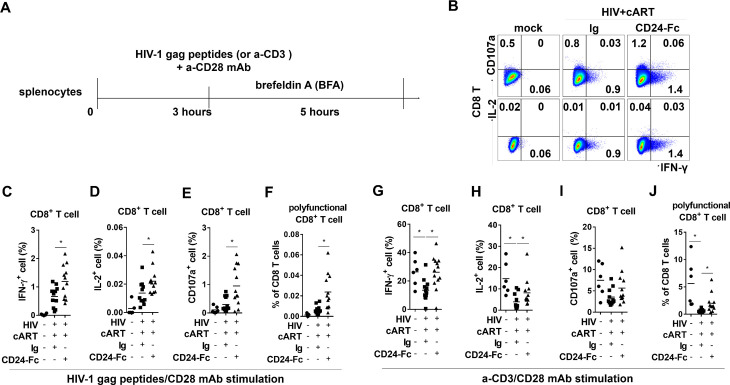
CD24-Fc treatment rescues CXCR5 + CD8 T cells and anti-HIV T cell response in vivo. **(A)** Splenocytes were stimulated ex vivo with either HIV gag peptides (B - F) or anti-CD3/CD28 antibodies **(G-J)**. **(B)** Representative plots for IFN-γ, IL-2 and CD107a expression in CD8 T cells after peptides stimulation. **(C)** Summary data for IFN-γ expression in CD8 T cells. **(D)** Summary data for IL-2 expression in CD8 T cells. **(E)** Summary data for CD107a expression in CD8 T cells. **(F)** Summary data for the percentage of IFN-γ + IL-2 + CD107a+ cells in CD8 T cells. **(G)** Summary data for IFN-γ expression in CD8 T cells. **(H)** Summary data for IL-2 expression in CD8 T cells. **(I)** Summary data for CD107a expression in CD8 T cells. **(J)** Summary data for the percentage of IFN-γ + IL-2 + CD107a+ cell in CD8 T cells. Bars represent mean values. *P* values calculated using the ordinary one-way ANOVA Turkey’s multiple comparisons test. * = *p* < 0.05.

To investigate whether CD24-Fc treatment could improve anti-HIV T cell responses, we conducted ex vivo splenocyte stimulation with HIV-1 gag peptides and anti-CD3/CD28 antibodies (Fig 3A). Cytokine analysis showed an enhanced viral-specific T cell response in splenocytes from CD24-Fc-treated mice, with increased numbers of IFN-γ or IL-2-producing cells compared to the cART + Ig group (Fig 3B – 3D). Additionally, CD8 + T cells from CD24-Fc-treated animals exhibited higher CD107a expression, indicating greater cytotoxic potential in HIV-1-specific CD8 + T cells (Fig 3E). HIV-specific CD8 + T cells in PWH often exhibit reduced polyfunctionality, correlated with the failure of viral control [51,52]. To identify HIV-1-specific polyfunctional T cells, we assessed IFN-γ, IL-2, and CD107a triple-positive cells upon stimulation with HIV peptides. The frequency of these triple-positive HIV-1-reactive CD8 + T cells was significantly higher in the CD24-Fc group than in the IgG control group (Fig 3F). Total T cell functionality was also improved with CD24-Fc, as shown by increased cytokine production T cells following anti-CD3/CD28 stimulation (Fig 3G – 3J). These results indicate that CD24-Fc treatment improves T cell functionality and anti-HIV responses in vivo, correlated with the restoration of CXCR5 + CD8 T_CM_ cells during suppressive cART.

### CD24-Fc treatment delays HIV-1 rebound and reduces viral pathogenesis after cART cessation

**Fig 4:**
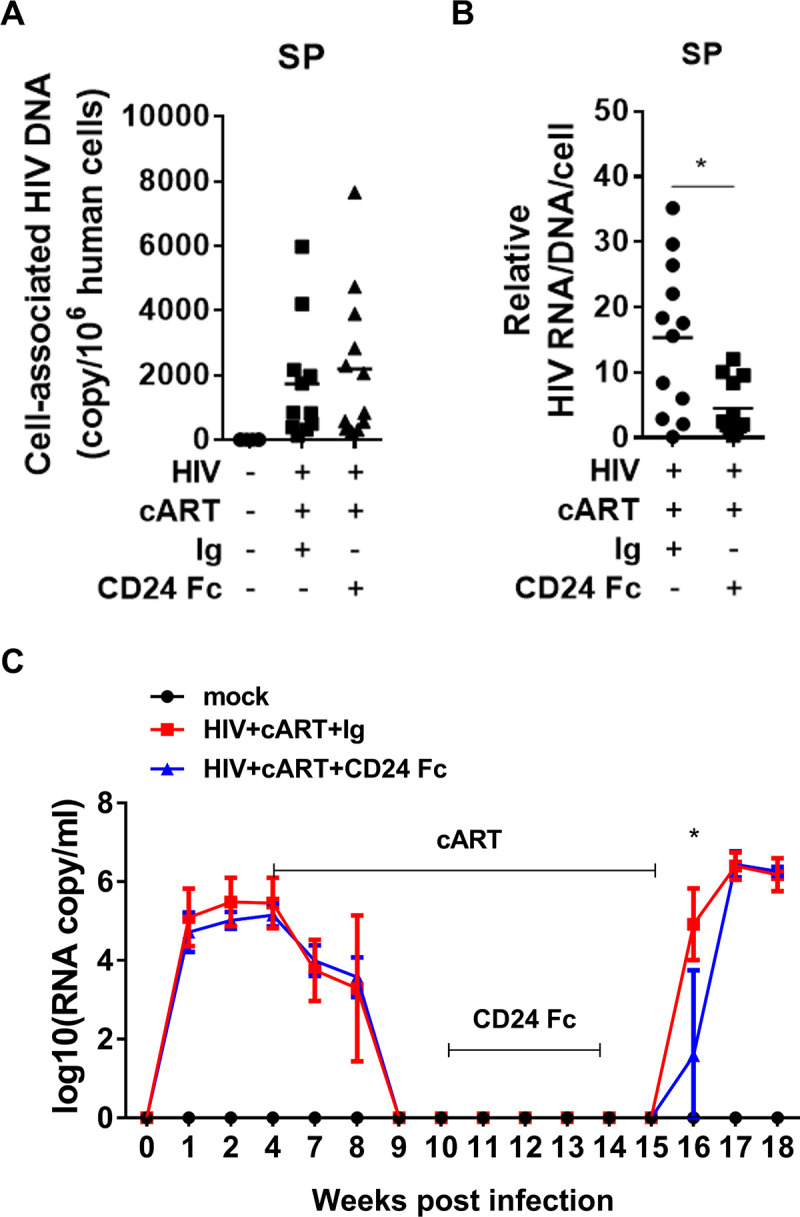
CD24-Fc treatment leads to reduced HIV gene expression during cART and delayed HIV-1 rebound after cART cessation. Humanized mice were infected with HIV-1 and treated as in Fig 1A. **(A)** The copy number of cell-associated HIV-1 DNA per million hCD45 + cells. **(B)** The ratio of HIV RNA over cell-associated HIV DNA to show relative HIV RNA/HIV genome. **(C)** Plasma HIV viral load with HIV rebound after cART cessation. Data shown is from two independent experiments. Bars represent mean values. *P* values calculated using two-tailed unpaired Mann-Whitney U-tests. * = *p* < 0.05. Error bars indicate s.e.m.

We hypothesized that the improved anti-HIV T cell response observed with CD24-Fc treatment during suppressive cART might control HIV reservoir and delay viral rebound after cART withdrawal. To test this, we assessed cell-associated HIV-1 DNA and RNA levels in splenocytes. Consistent with the lack of HIV reservoir activation during CD24-Fc treatment, we observed no significant difference in cell-associated HIV-1 DNA between the IgG and CD24-Fc groups (Fig 4A). However, CD24-Fc treatment reduced the HIV-1 RNA/DNA ratio compared to the IgG group, indicating that viral gene expression in reservoir cells was reduced by CD24-Fc treatment (Fig 4B). To explore if CD24-Fc treatment could delay HIV-1 rebound, we conducted an HIV-1 rebound experiment, stopping cART eight days after the final CD24-Fc injection. Viral load analysis showed a one-week delay in viral rebound in the CD24-Fc group compared to the IgG group (Fig 4C). All animals were euthanized at 18 wpi when viremia stabilized in all animals. No significant differences in human cell number (S2A-S2C Figs) or T cell activation (S2D Fig) were observed between CD24-Fc and Ig-treated groups in splenocytes.

**Fig 5:**
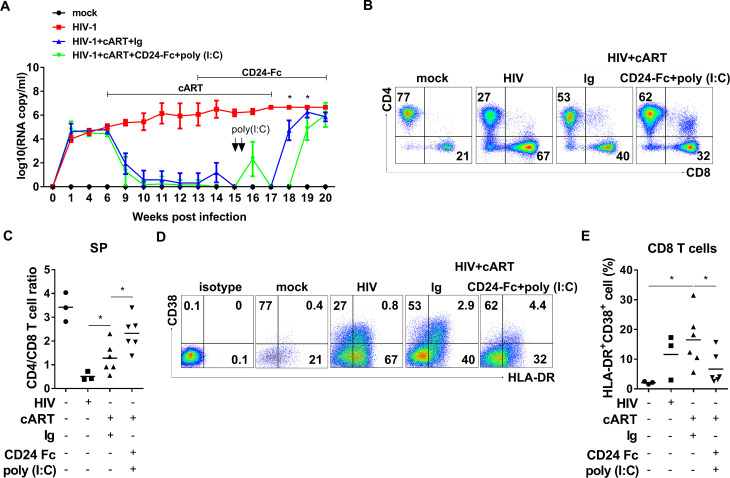
CD24-Fc treatment and HIV latency reversal delay HIV-1 rebound and reduce HIV pathogenesis after cART cessation. Humanized mice were infected with HIV, starting with cART from 6 wpi to 17wpi. CD24-Fc treatment introduced at 13 wpi until termination. Ploy(I:C) was administered at 15 wpi. All animals were terminated at 20 wpi. **(A)** Viremia detected at the indicated time points with treatments indicated. **(B)** Representative FACS plots show the frequency of CD4+ and CD8 + T cells. **(C)** Summary graphs of CD4 and CD8 T cell ratios. **(D)** Representative FACS plots show the frequency of HLA-DR + CD38 + cells in CD8 + T cells. **(E)** The percentage summary of HLA-DR + CD38 + cells in CD8 T cells. Bars represent mean values. *P* values calculated using the ordinary one-way ANOVA Turkey’s multiple comparisons test. * = *p* < 0.05. Error bars indicate mean values ± s.e.m.

Since viral reservoirs were not reactivated during CD24-Fc and cART treatment, reservoir cells likely evaded targeting by HIV-specific cytotoxic lymphocytes (CTLs). To enhance therapeutic efficacy, we added two doses of poly (I:C), known to both reactivate HIV-1 replication and enhance anti-HIV immunity but by itself does not reduce HIV-1 reservoir or HIV-1 rebound [40], during CD24-Fc and cART treatments. Additionally, we extended CD24-Fc treatment post-cART cessation until the experiment’s end to leverage its anti-inflammatory effects after HIV rebound (Fig 5A). Remarkably, in the CD24-Fc + poly (I:C) group, HIV viremia rebound was delayed by approximately two weeks compared to the IgG group. Viremia levels in all infected mice reached a similar point three weeks post-cART withdrawal, at which point all animals were euthanized (Fig 5A). In the spleens at termination, the CD24-Fc+poly (I:C) group showed a higher CD4 + /CD8 + T cell ratio compared to the IgG group, which only partially increased the CD4/CD8 ratio relative to mock and HIV-only groups (Fig 5B and 5C). Additionally, CD24-Fc+poly (I:C) treatment decreased the frequency of activated (HLA-DR + CD38+) CD8 T cells compared to the IgG group (Fig 5D and 5E). No significant differences in human leukocyte and T cell numbers in the spleen were noted between IgG and CD24-Fc + poly (I:C) groups (S3 Fig). Therefore, CD24-Fc treatment during suppressive cART provided multiple benefits, including reduction of HIV-1 immune pathogenesis, enhanced anti-HIV T cell responses and delayed viral rebound following cART cessation in humanized mice.

### CD24-Fc increases CXCR5 + CD8 T cells and anti-HIV T cells in PBMC from PWH in vitro

**Fig 6:**
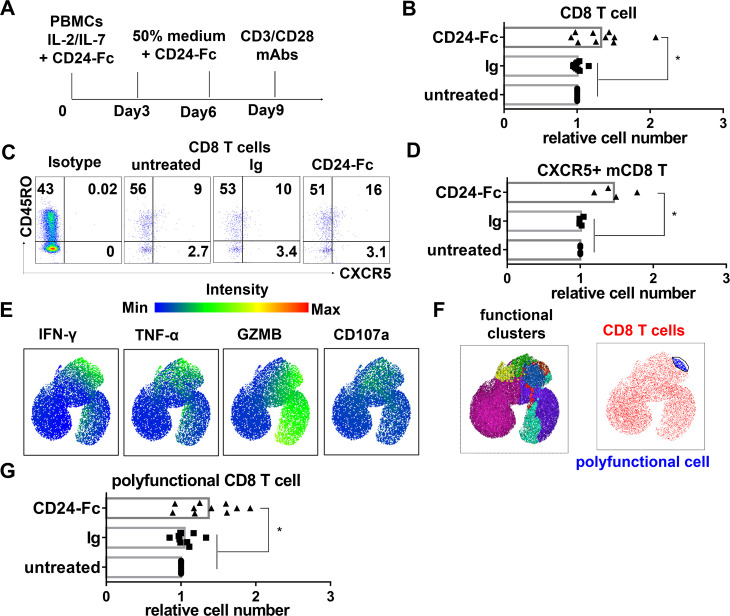
CD24-Fc treatment increases CXCR5 + memory CD8 T cell and T cell functionality in PBMCs from PWH in vitro. **(A)** Schematic description of experiment. PBMCs from PWH were cultured with CD24-Fc for 9 days and stimulated with anti-CD3/CD28 antibodies after culture. **(B)** The relative number of CD8 T cells at the end of culture. **(C)** Representative CYTEK plots show the frequency of CXCR5 + memory cell in CD8 T cells. **(D)** Summary graph for the relative number of CXCR5 + memory CD8 T cells. **(E)** Expression of different effector markers in CD8 T cells by UMAP based on 23-color CYTEK Spectral flow cytometry. **(F)** Polyfunctional cluster (4 effector markers) identified by FlowSOM software and polyfunctional T cells (blue) over total CD8 T cells (red). **(G)** Relative numbers of polyfunctional CD8 T cells in individual donors in different groups. Data shown are from two independent experiments. Bars represent mean values. *P* values calculated using two-tailed T-tests. * = *p* < 0.05.

To evaluate the effects of CD24-Fc on immune cells from PWH, we cultured peripheral blood mononuclear cells (PBMCs) from virologically suppressed PWH. Cells were treated in vitro with or without CD24-Fc protein for 9 days before assessing T cell functionality by multi-color CYTEK spectral cytometry (Fig 6A). First, we compared the number and function of CD8 + T cells between HC and PWH after culture. We found that more viable CD8 T cells from HC than PWH, produced relatively more IFN-γ or TNF-α following upon anti-CD3/CD28 stimulation (S4A and S4B Figs). Focusing on CD8 + T cells producing all 4 effectors (IFN-γ, TNF-α, granzyme B, and CD107a) simultaneously (S4C and S4D Figs), we found that PWH CD8 + T cells consisted of fewer polyfunctional CD8 T cells than HC cells (S4E Fig). CD24-Fc treatment increased the number of CD8 + T cells in PWH cultures (Fig 6B), including the number of CXCR5 + memory CD8 T cells (Fig 6C and6D), though their relative frequency in total memory CD8 + T cell population was not significantly elevated by CD24-Fc (S5A Fig). In PBMC cultures from PWH, CD24-Fc treatment led to an increase in the number and frequency of CD8 T cells expressing effector markers such as IFN-γ, TNF-α, granzyme B, and CD107a, compared to control groups (S5B and S5C Figs). By auto-clustering analysis of CD8 + T cells expressing all 4 effectors, we further analyzed polyfunctional CD8 + T cells (Fig 6E and6F). The number of polyfunctional CD8 + T cells was increased significantly by CD24-Fc treatment (Fig 6G), although the frequency in total CD8 T cells was not significantly elevated (S5D Fig).

## Discussion

HIV-1 reservoirs in people with HIV (PWH) contribute to chronic inflammation-associated diseases. We tested the therapeutic effect of the anti-inflammatory fusion protein CD24-Fc in humanized mice with HIV-1 infection and suppressive cART in vivo and in PMBC from PWH with cART in vitro. We report here that CD24-Fc reduced chronic inflammation and HIV-1 immune pathogenesis, enhanced CD8 memory T cells with antiviral functions in humanized mice with HIV and cART, and in PWH PBMCs. CD24-Fc delayed virus rebound and reduced viral pathogenesis after cART cessation. Our findings indicate that CD24-Fc could provide a novel therapeutic for treating HIV-associated chronic inflammation and its associated diseases in PWH.

Persistent inflammation and immune hyper-activation play critical roles in HIV-1 associated diseases in post cART era. Over 30% of PWH who initiate cART with low CD4 counts experience poor CD4 T cell reconstitution [4–8], with greater immune hyper-activation and inflammation compared to those with better CD4 recovery [9], and at an elevated risk for comorbidities and non-AIDS events [10–12]. We discovered that CD24-Fc treatment reduced chronic inflammation and enhanced CD4 T cell recovery, associated with reduced inflammatory cytokines including IFN-I, TNFα, IP10, MCP-1 and IL-8. In addition, CD24-Fc also enhanced CD8 memory T cells with antiviral functions in humanized mice with HIV and cART, and in PWH PBMCs. Furthermore, CD24-Fc delayed virus rebound and reduced viral pathogenesis after cART cessation. Targeting residual inflammation in PWH under cART with the CD24-FC protein may offer a promising therapeutic avenue for managing HIV-1 and related diseases.

We observe that CD24-Fc increased central memory CD8 T cells (including CXCR5 + memory T cells) in humanized mice in vivo or PBMC from PWH in vitro, associated with elevated anti-HIV T cell response. CXCR5 + memory CD8 + T cells have been reported to exhibit more potent cytotoxicity than the CXCR5 − subset [50,53]. We will investigate the effect of CD24-Fc on differentiation, proliferation and maintenance of CD8 memory T cells and their antiviral activity in future study. In addition, we anticipate that a combination immunotherapy involving CD24-Fc, PD-1/PD-L1 checkpoint inhibitors, and latency-reversing agents (LRAs) could further boost the anti-HIV immune response, reduce HIV reservoirs and delay or prevent viral rebound upon cART cessation.

It is important to point out that the human immune system reconstituted in NSG-hu HSC mice, though with functional human immune cells such as T cells, plasmacytoid dendritic cells (pDC) and conventional DC [41,54–56], shows major defects in low monocytes and macrophages [57]. We and others have reported that HIV-1 infection or adjuvants lead to elevated monocytes and macrophages and HIV-related inflammatory diseases [38,40,41,44,58]. Interestingly, pathogenic human macrophages are induced in humanized mice infected with HIV-1 under cART [59]. It is important that the key findings in humanized mice be confirmed in human patients. We thus used PBMCs from PWH to confirm that CD24-Fc also increased human T cells with anti-HIV functions in vitro assay. As CD24-Fc has been used in human subjects with no safety concerns, these findings provide supporting evidence to test CD24-Fc in PWH in future clinical trials.

It has been reported that CD24 interacts with Siglec-10 (or Siglec-G in mice) to repress inflammatory response to danger-associated molecular patterns (DAMPs) in human or mice, respectively [60]. Inflammatory diseases with chronic cell death generate DAMPs to induce inflammatory responses. The recombinant fusion protein CD24-Fc was developed to suppress inflammation and immune-related adverse events through binding to and sequestering DAMPs and interacting with the Siglec-G/10 receptor [24,60]. The recent phase II clinical trial in patients who undergo human leukocyte antigen–matched unrelated donor allogeneic hematopoietic stem cell transplantation, CD24-Fc treatment has been shown to significantly prevent acute graft-versus-host disease (GVHD development with no unexpected drug-related immunotherapy-related adverse events (irAEs) or toxicities observed [34]. In hospitalized COVID-19 patients who needs oxygen support, CD24-Fc is well tolerated and the treatment significantly accelerates clinical improvement and systematically repress inflammatory response in patients [35,61]. It is recently reported that CD24 expressed on tumor cells can exert a “don’t eat me” signal when binding to Siglec-10 on macrophages, which could impede macrophage phagocytosis activity. [62,63]. Interestingly, CD24-Fc ameliorates irAEs when used in combination with immune checkpoint inhibitors in tumor immunotherapy in humanized NSG mice engrafted with human hematopoietic stem cells [63].

HIV infection induces depletion of immune cells through both direct cytopathic effect in infected CD4 + T cells [14,64–66] or indirectly by inflammation-induced cell death [67–69]. HIV-associated cell death may activate inflammation through releasing danger-associated molecular patterns (DAMPs) molecules such as high mobility group box 1 (HMGB1) [70–72]. HMGB1 is known to facilitate inflammation and induce immune suppression in chronic inflammatory diseases [73]. In SIV-infected non-human primates without cART, CD24-Fc is able to reduce inflammation, alleviate chronic immune activation and slow down AIDS progression [32,33]. In this report, we show that CD24-Fc treatment enhanced human CD4 T cell recovery and anti-HIV CD8 + T cell immune function and control of viral reservoirs. Our results indicate that CD24-Fc therapy, combined with cART, significantly reduced HIV-associated inflammation and immune activation in HIV-1 infected humanized mice. Importantly, CD24-Fc elevated CD8 + T cells with anti-HIV polyfunctionality, correlated with CXCR5 + memory CD8 + T cells and delay of virus rebound after cART cessation.

In humans, Siglec-10 is predominantly expressed on dendritic cells, natural killer (NK) cells, and B cell [74]. It is reported that activated CD4 + T cells upregulate Siglec-10 expression [75], which is associated with persistent abnormal CD4 T cell activation in PWH despite viral suppression by cART [76]. In this study, CD24-Fc treatment appeared to enhance CD4 T cell recovery in vivo. This improvement may arise either from a direct effect on CD4 T cells or indirectly via systemic inflammation reduction due to its broader anti-inflammatory effects.

## Materials and methods

### Ethics statement

All animal experiments were reviewed and approved by the Institutional Animal Care and Use Committee (IACUC) at the University of North Carolina at Chapel Hill (Protocol ID: 16–073).

### Humanized mice

Humanized mice were generated as previously described [31,43,55,56,77,78]. Briefly, NOD-Rag1^null^IL2rg^null^ (NRG) neonates (1-to-5 days old) were irradiated (250 rads) and injected with 2 x 10⁵ human CD34 + hematopoietic stem cells (HSCs) into the liver. HSCs were isolated from human fetal liver tissues obtained from elective or medically indicated pregnancy terminations through a non-profit intermediary working with outpatient clinics (Advanced Bioscience Resources).

### HIV-1 infection of humanized mice

Humanized mice were infected via retro-orbital injection with HIV-1_JRCSF_ stocks (10 ng p24/mouse) or 293T mock transfection supernatants for control mice.

### cART regimens in humanized mice

Individual tablets of TRUVADA (tenofovir/emtricitabine; Gilead Sciences) or raltegravir (Merck) were crushed into fine powder and manufactured as 5BXL by TestDiet based on previously published [29,38,79].

### CD24-Fc Fusion Protein Treatment

CD24-Fc protein was obtained from Yang Liu’s lab as a gift. The recombinant CD24-Fc fusion protein and IgG-Fc was manufactured according to current ideal manufacturing procedures as previously described [33]. Humanized mice were administered twice a week with 200μg recombinant protein each dosage through intraperitoneal injection (i.p.).

### HIV viral load in plasma

Blood was collected by tail vein bleeding using EDTA as an anticoagulant, and plasma was stored at -80°C until assay. HIV-1 RNA was extracted from plasma using the Viral RNA Mini Kit (Qiagen) and quantified by real-time PCR with the TaqMan Fast Virus One Step PCR kit (ThermoFisher Scientific) on a QuantStudio 6 Flex PCR system (Applied Biosystems), with a detection limit of 400 copies/ml [29,31,38,43].

### Real-time PCR

For detecting interferon-stimulated genes (ISGs), RNA was isolated from splenocytes using the RNeasy Plus extraction kit (Qiagen) and converted to cDNA using SuperScript III First-Strand Synthesis (Invitrogen). ISG levels in cDNA were quantified by real-time PCR with human gene-specific primers as previously described [29,30].

For cell-associated HIV-1 DNA, nucleic acid was extracted from cells or tissues using DNeasy mini kit (Qiagen). HIV-1 DNA was quantified by real-time PCR. Genomic DNA of ACH2, which contains one copy of HIV genome in each cell, was serially diluted in mouse leukocytes DNA to generate a standard curve [29].

For cell-associated HIV-1 RNA, RNA was extracted from cells or tissues using RNeasy plus mini kit (Qiagen). HIV-1 RNA was detected as previously described [29,31,43]. The HIV-1 gag RNA expression was normalized to human CD4 mRNA level and relative HIV-1 gene expression levels were calculated according to 2^-ΔΔCT^ [29,80].

### Anti-HIV T cells detection

Cells from humanized mice spleen were stimulated ex vivo with an HIV gag peptide pool (2 μg/ml per peptide; PepMix HIV (GAG) Ultra, JPT Innovation Peptide Solutions) and human CD28 antibody (2 μg/ml) for 3 hours without, and then 5 hours with, brefeldin A. Cells were fixed, permeabilized, and subjected to intracellular staining.

### Human PBMC samples

Human PBMC samples were kindly provided by Dr. Poonam Mathur and Dr. Shyamasundaran Kottilil (University of Maryland, Baltimore). PBMC samples were collected from HIV-infected participants who were on cART for at least 12 months, and plasma HIV RNA levels were ≤ 40 copies/ml, as measured by the Abbott Real-Time HIV-1 PCR at the time of sample collection.

### In vitro human PBMC assays

PBMCs from PWH or HIV-negative donors were cultured at 1 x 10⁶ cells/ml in 10% FBS RPMI-1640 containing 20 U/ml IL-2, 10 ng/ml IL-7, and CD24-Fc protein (10 μg/ml) or IgG (10 μg/ml). Controls received no treatment. Every 3 days, 50% of the medium was replaced with fresh medium containing 40 U/ml IL-2, 20 ng/ml IL-7, and 20 μg/ml CD24-Fc protein. On day 9, live cells were counted and cultured in complete medium with anti-CD3 (1 μg/ml, clone 30-F11; Biolegend) and anti-CD28 (1 μg/ml, clone CD28.2; Biolegend) antibodies. Cells were stained for flow cytometry after 6 hours of incubation with brefeldin A at 37°C.

### Flow cytometry and data analysis

For intracellular staining, cells were stained with surface markers first, and then permeabilized with cytofix/cytoperm buffer (BD Bioscience, cat#554714), followed by intracellular staining. Anti-human antibodies were purchased from Biolegend, including anti-CXCR5 (clone:J252D4), anti-CD4 (clone:RP4-T4), anti-CD8 (clone:HIT8a), anti-CD3 (clone:HIT3a), anti-CD45 (clone:H130), anti-CD45RA (clone:H100), anti-CCR7 (clone:G043H7), anti-KLRG1 (clone:SA231A2), anti-CD161 (clone:W18070C), anti-CD57 (clone:QA17A04), anti-HLA-DR (clone:L243), anti-CD38 (clone:HIT2), anti-PD-1 (clone:NAT105), anti-IFN-γ (clone:4S.B3), anti-TNF-α (clone: Mab11), granzyme B (clone: GB11), CD107a (H4A3) and anti-IL-2 (clone:MQ1-17H12). Anti–mouse CD45 (clone: HI30) and LIVE/DEAD Fixable Aqua Dead Cell Stain Kit (cat#L34957) were purchased from Invitrogen. Flow cytometry was performed using multi-color CYTEK spectral cytometry and analyzed by FlowJo software version 10.8.1 (FlowJo LLC).

### UMAP analysis

Dimensionality reduction was performed using the UMAP. For UMAP analysis, live CD3 + CD4-CD8 + populations were concatenated. UMAP plots were generated with default settings and excluding all parameters used upstream in the gating strategy (CD3, CD4 and CD8). The same numbers of CD8 + T cells from HIV negative and HIV positive individuals were similarly applied to UMAP analysis. Markers considered in data from humanized mice include CD45RA, CCR7, CXCR5, PD-1, CD28, CD57, CD161 and KLRG1. Markers considered in data from humanized mice include CD45RA, CCR7, PD-1, CD28, CD57, CD161 and KLRG1. Markers considered in data from humanized mice include CD45RA, CCR7, PD-1, CXCR5, PD-1, IFN-γ, IL-2, CD107a, granzyme B. We identify polyfunctional cluster expressing multi T cell effector markers using FlowSOM version 4.0.0 plugin. ClusterExplorer version 1.7.6 plugin was used to map clusters on UMAP plot. To characterize cell cluster, manual gating was applied on UMAP space based on the auto-clusters, and then pseudo-colored was applied on the UMAP plot.

### Statistical analysis

All experiments were analyzed using Prism 7 (GraphPad Software). Statistical differences were assessed using two-tailed unpaired T-tests or two-tailed unpaired Mann-Whitney U-tests, or Ordinary one-way ANOVA Turkey’s multiple comparisons test. P values of <0.05 indicated the significant difference between relevant groups.

## Supporting information

S1 Fig Related to Fig 2. CD24-Fc treatment reduces HIV-1 pathogenesis during cART. Humanized mice were infected and treated as in Fig 1A and splenocytes were analyzed by FACS on termination. (A) Representative FACS plots show the frequency of CD4+ or CD8 + T cell in CD3 + cells. (B) Summary graphs show the number of hCD45 + cell, CD4 + T, CD8 + T cell and CD3- cell per spleen. (C) Representative FACS for the expression of HLA-DR and CD38 expression in CD8 T cells. (D) Representative FACS for the expression of CD45RA and CCR7 expression in CD8 T cells. (E) Summary graphs show the frequency of PD-1 + cell in central memory or effector memory CD8 T cells. (F) Summary graphs show the frequency of CXCR5 + cell in total CD8 T cells. Bar represents mean value.
(TIF)

S2 Fig Related to Fig 4. CD24-Fc treatment during cART does not rescue human immune cells after cART cessation and virus rebound. Humanized mice were infected and treated as in S5E Fig. (A) Human CD45 + cell number. (B) CD4 T cell number per spleen. (C) CD8 T cell number per spleen (D) The frequency of HLA-DR and CD38 double positive CD8 T cells. Bar represents mean value.
(TIF)

S3 Fig Related to Fig 5. Sustained CD24-Fc treatment after cART cessation and virus rebound rescues human immune cells. Humanized mice were infected and treated as in Fig 5A and splenocytes were analyzed by FACS. Summary graphs show the number of human CD45 + cell, CD4 + T cell or CD8 + T cell per spleen. Bar represents mean value. *P* values calculated using ordinary one-way ANOVA Turkeys multiple comparisons test. * = p < 0.05.
(TIF)

S4 Fig Related to Fig 6. The functionality of CD8 T cells is impaired by HIV infection. PBMCs from HC or PWH were culture for 9 days as in Fig 7 without treatment. (A) CD8 T cell number. (B) Relative number of individual marker positive CD8 T cells. (C) Polyfunctional cluster identified by FlowSOM and polyfunctional cells gated on accordingly (blue). (D) Histograms show individual cytokine expression intensity in polyfunctional cell (blue) over the intensity of total CD8 T cells (red). (E) The relative number of polyfunctional CD8 T cell in individual donor in different groups after culture. Bar represents mean value. *P* values calculated using two-tailed unpaired Mann-Whitney U-tests. * = *p* < 0.05.
(TIF)

S5 Fig Related to Fig 6. CD24-Fc treatment rescues T cell function in PWH PBMCs in vitro. (A) The frequency of CXCR5 + memory cell in memory CD8 T cells. (B) The relative number of individual marker positive cell number. (C) The frequency of individual marker positive cell in CD8 T cells. (D) The frequency of polyfunctional CD8 T cell as in Fig 6G. Bar represents mean value. *P* values calculated using two-tailed unpaired Mann-Whitney U-tests. * = p < 0.05
(TIF)

S1 Text Support information for Fig 2A. The raw value in each group for making Fig 2A. Support information for Fig 2B: The raw value in each group for making Fig 2B. Support information for Fig 2C: The raw value in each group for making Fig 2C. Support information for Fig 2E: The raw value in each group for making Fig 2E. Support information for Fig 6B: The raw value in each group for making Fig 6B. Support information for Fig 6D: The raw value in each group for making Fig 6D. Support information for Fig 6G: The raw value in each group for making Fig 6G.
(DOCX)

S1 Table Information about humanized mice (Related to Figs 1–3).
(DOCX)
