# Supplementary figures and images for: CD24-Fc resolves inflammation and enhances anti-HIV CD8 T cells with polyfunctionality during HIV-1 infection under cART
Source: PLoS Pathog. 2025 Aug 8;21(8):e1012826. doi: 10.1371/journal.ppat.1012826 (PMC12349878; doi:10.1371/journal.ppat.1012826)

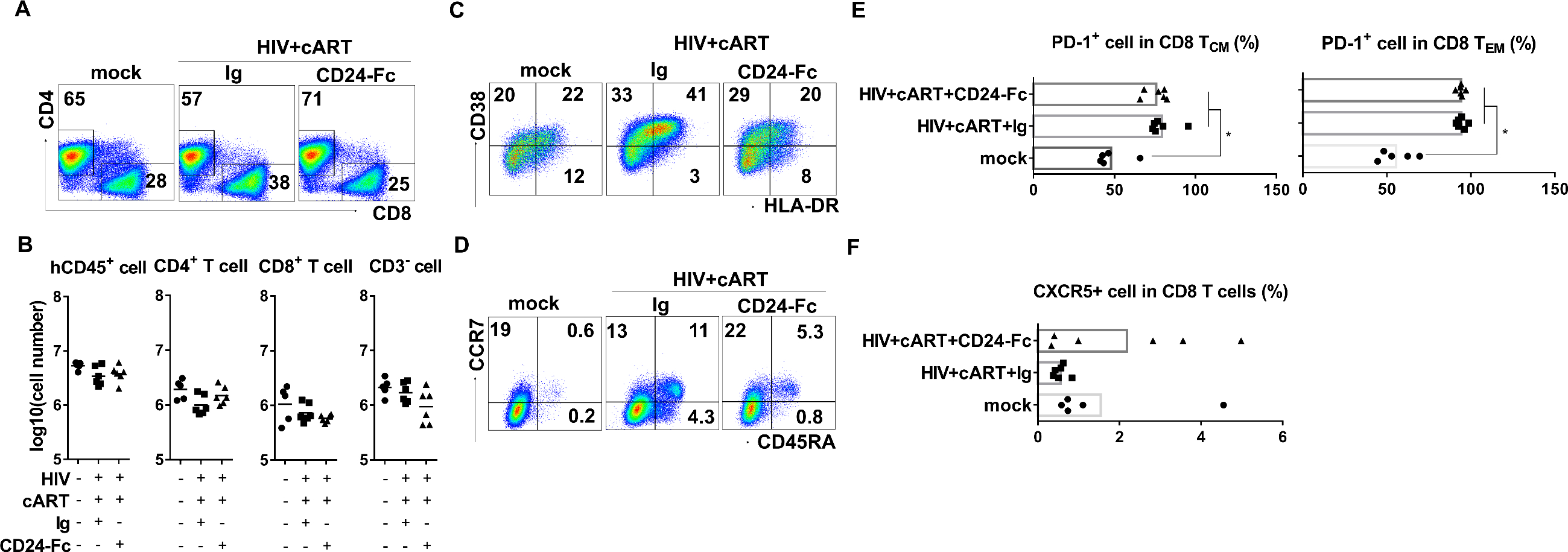

Supplement: S1 Fig — Humanized mice were infected and treated as in Fig 1A and splenocytes were analyzed by FACS on termination. (A) Representative FACS plots show the frequency of CD4+ or CD8 + T cell in CD3 + cells. (B) Summary graphs show the number of hCD45 + cell, CD4 + T, CD8 + T cell and CD3- cell per spleen. (C) Representative FACS for the expression of HLA-DR and CD38 expression in CD8 T cells. (D) Representative FACS for the expression of CD45RA and CCR7 expression in CD8 T cells. (E) Summary graphs show the frequency of PD-1 + cell in central memory or effector memory CD8 T cells. (F) Summary graphs show the frequency of CXCR5 + cell in total CD8 T cells. Bar represents mean value. (TIF) [file ppat.1012826.s001.tif]

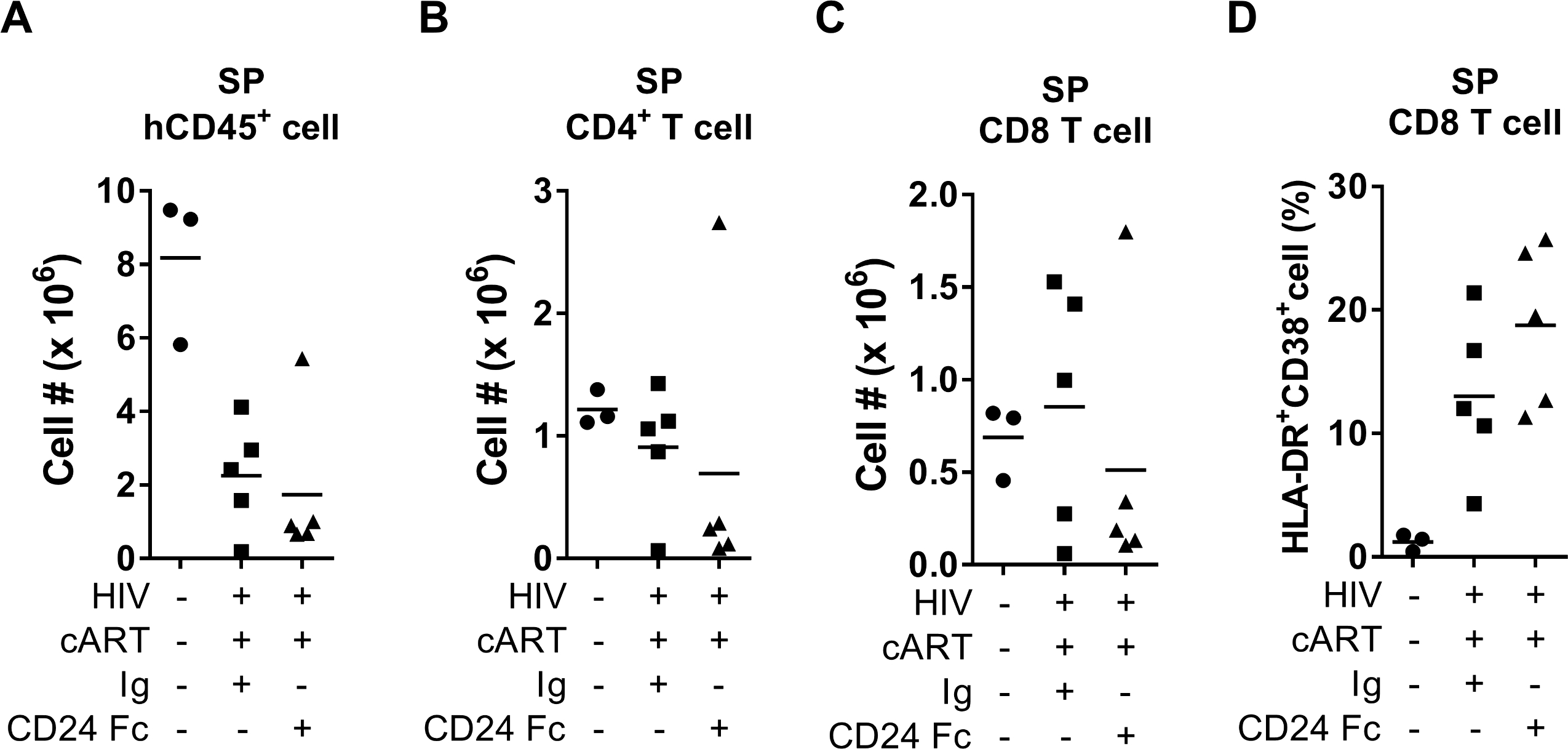

Supplement: S2 Fig — Humanized mice were infected and treated as in S5E Fig. (A) Human CD45 + cell number. (B) CD4 T cell number per spleen. (C) CD8 T cell number per spleen (D) The frequency of HLA-DR and CD38 double positive CD8 T cells. Bar represents mean value. (TIF) [file ppat.1012826.s002.tif]

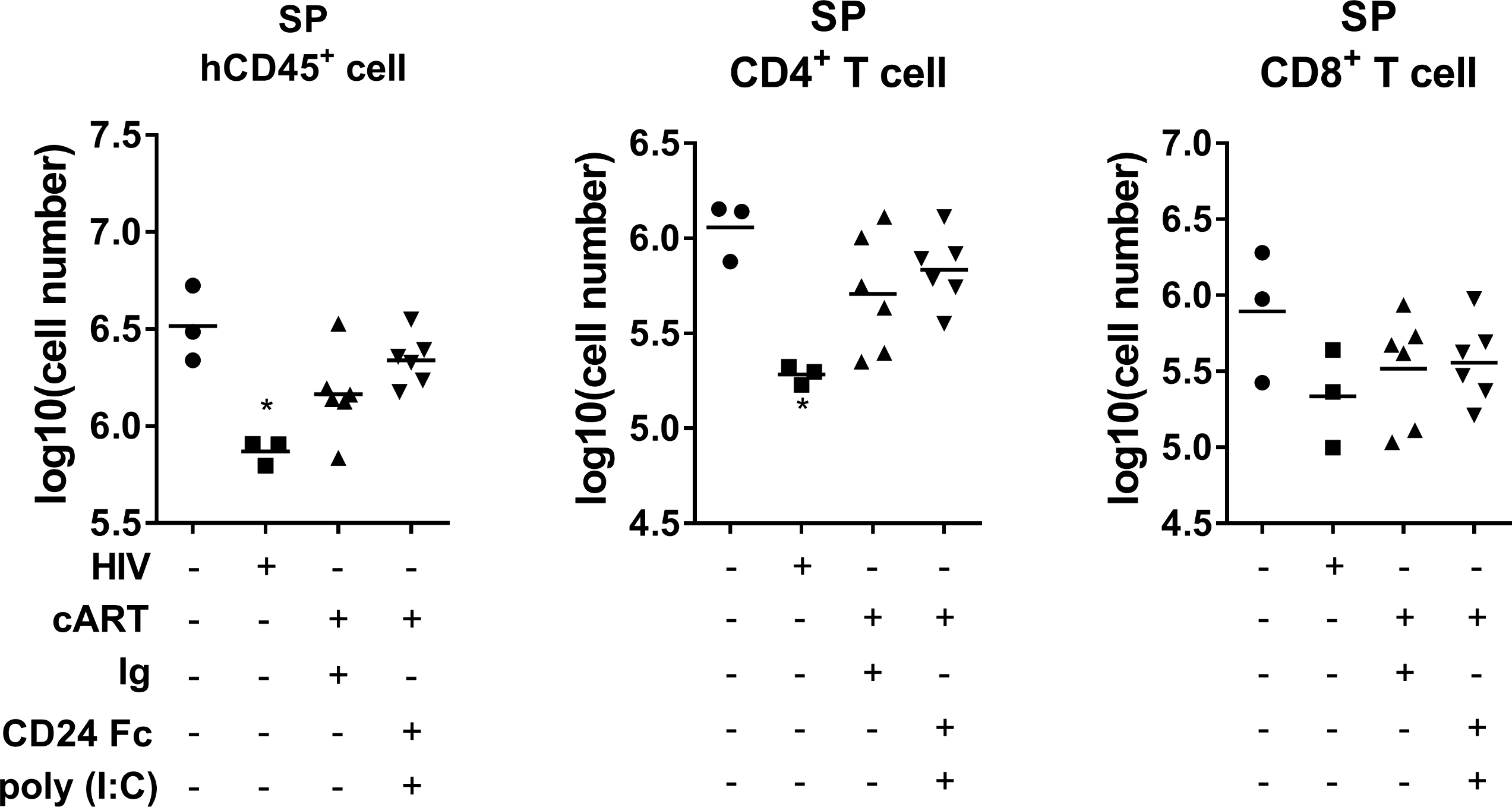

Supplement: S3 Fig — Humanized mice were infected and treated as in Fig 5A and splenocytes were analyzed by FACS. Summary graphs show the number of human CD45 + cell, CD4 + T cell or CD8 + T cell per spleen. Bar represents mean value. P values calculated using ordinary one-way ANOVA Turkeys multiple comparisons test. * = p < 0.05. (TIF) [file ppat.1012826.s003.tif]

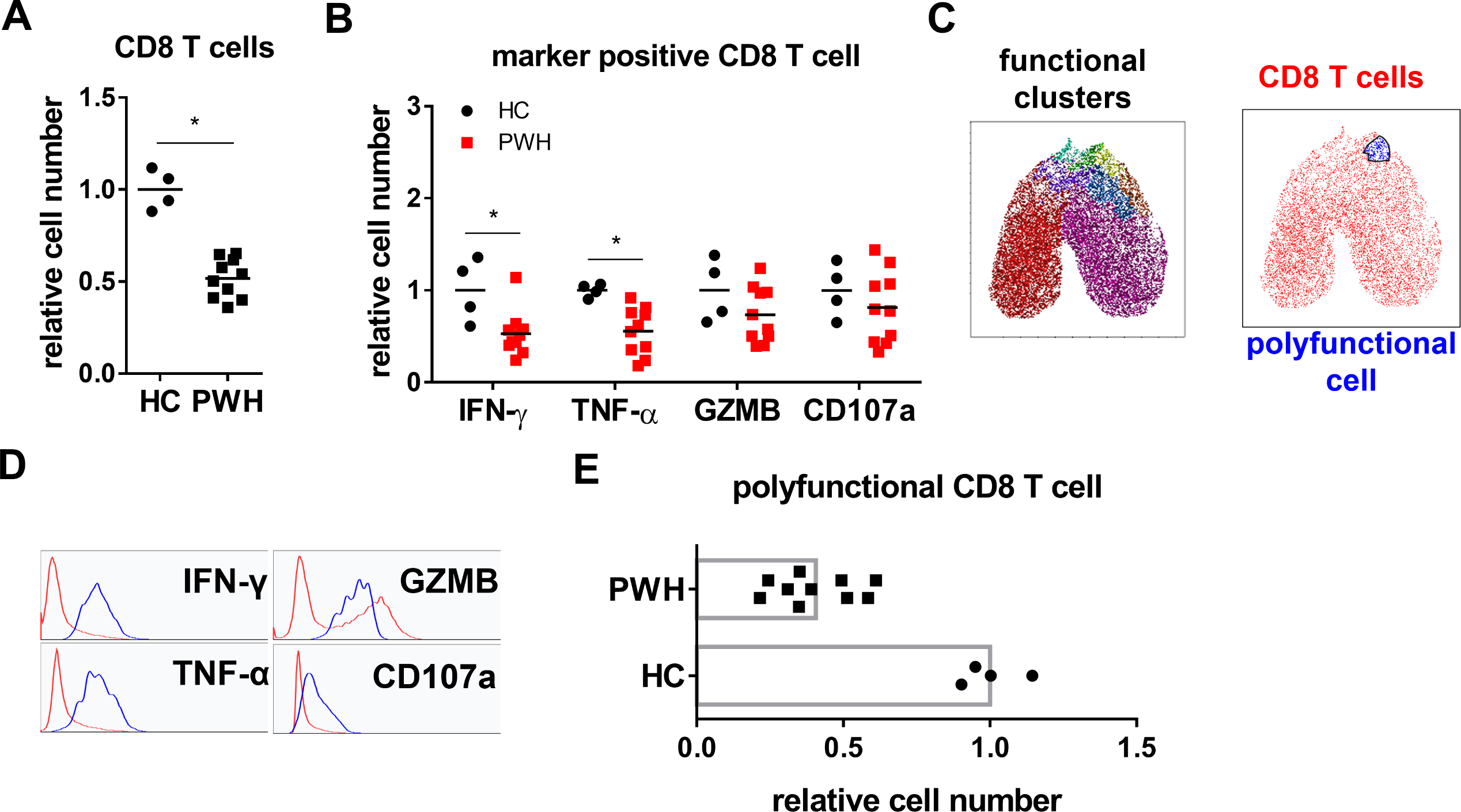

Supplement: S4 Fig — PBMCs from HC or PWH were culture for 9 days as in Fig 7 without treatment. (A) CD8 T cell number. (B) Relative number of individual marker positive CD8 T cells. (C) Polyfunctional cluster identified by FlowSOM and polyfunctional cells gated on accordingly (blue). (D) Histograms show individual cytokine expression intensity in polyfunctional cell (blue) over the intensity of total CD8 T cells (red). (E) The relative number of polyfunctional CD8 T cell in individual donor in different groups after culture. Bar represents mean value. P values calculated using two-tailed unpaired Mann-Whitney U-tests. * = p < 0.05. (TIF) [file ppat.1012826.s004.tif]

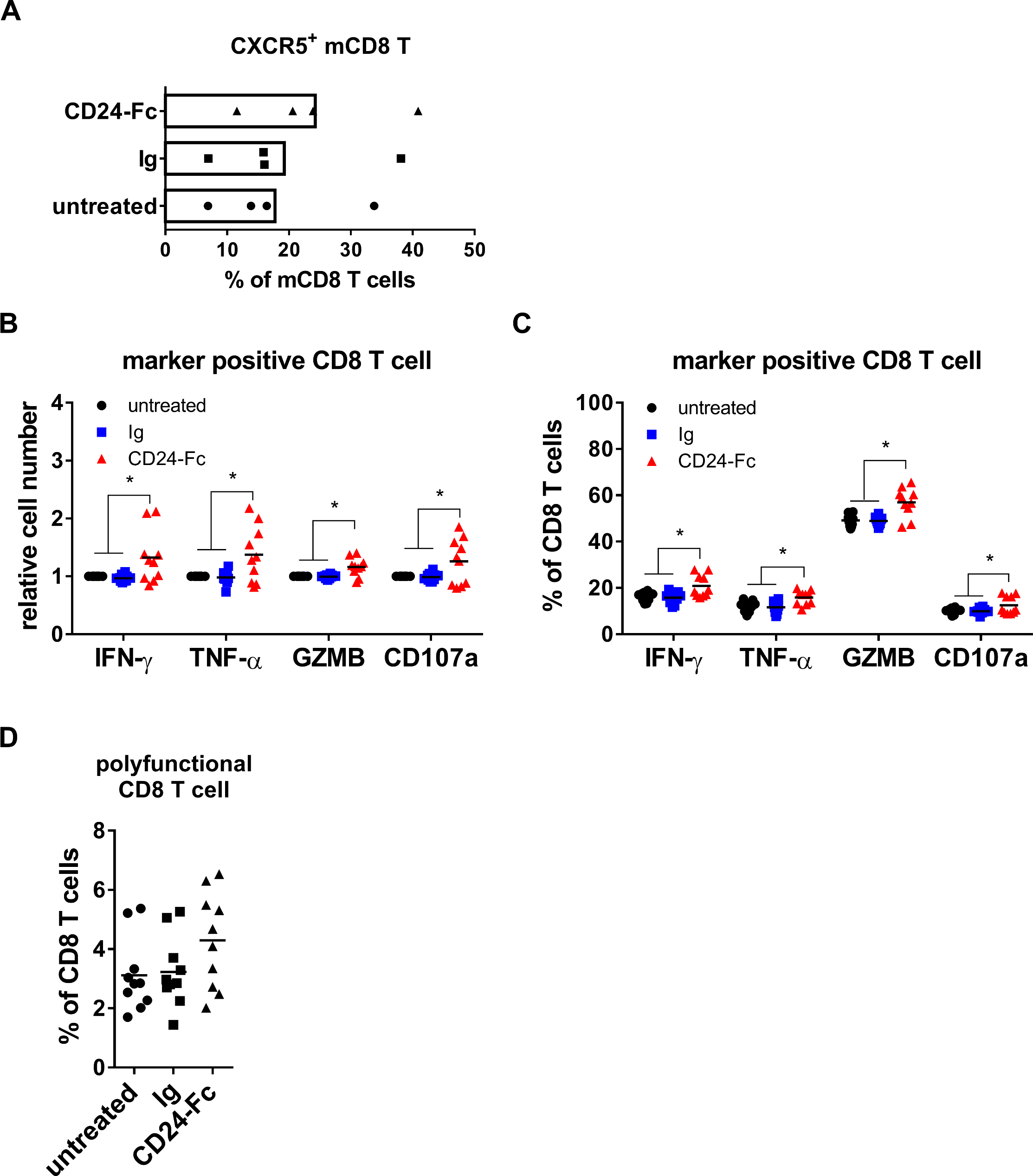

Supplement: S5 Fig — (A) The frequency of CXCR5 + memory cell in memory CD8 T cells. (B) The relative number of individual marker positive cell number. (C) The frequency of individual marker positive cell in CD8 T cells. (D) The frequency of polyfunctional CD8 T cell as in Fig 6G. Bar represents mean value. P values calculated using two-tailed unpaired Mann-Whitney U-tests. * = p < 0.05 (TIF) [file ppat.1012826.s005.tif]
