# Supplementary material for: CD24-Fc resolves inflammation and enhances anti-HIV CD8 T cells with polyfunctionality during HIV-1 infection under cART
Source: PLoS Pathog. 2025 Aug 8;21(8):e1012826. doi: 10.1371/journal.ppat.1012826 (PMC12349878; doi:10.1371/journal.ppat.1012826)
Supplement: S1 Text — Support information for Fig 2A. The raw value in each group for making Fig 2A. Support information for Fig 2B: The raw value in each group for making Fig 2B. Support information for Fig 2C: The raw value in each group for making Fig 2C. Support information for Fig 2E: The raw value in each group for making Fig 2E. Support information for Fig 6B: The raw value in each group for making Fig 6B. Support information for Fig 6D: The raw value in each group for making Fig 6D. Support information for Fig 6G: The raw value in each group for making Fig 6G. (DOCX) [file ppat.1012826.s006.docx]

| Fig 2A | | |
| --- | --- | --- |
| mock | HIV+cART+Ig | HIV+cART+CD24-Fc |
| 1.64 | 1.43 | 1.53 |
| 1.39 | 1.35 | 3.02 |
| 3.11 | 1.23 | 2.38 |
| 1.53 | 0.68 | 4.08 |
| 2.37 | 1.45 | 2.48 |
|  | 1.31 | 2.63 |

| Fig 2B | | |
| --- | --- | --- |
| mock | HIV+cART+Ig | HIV+cART+CD24-Fc |
| 20.40 | 21.20 | 6.59 |
| 22.50 | 28.70 | 11.50 |
| 20.50 | 22.80 | 8.17 |
| 8.88 | 41.00 | 7.86 |
| 14.30 | 22.80 | 23.40 |
|  | 47.70 | 22.50 |

| Fig 2C | | |
| --- | --- | --- |
| mock | HIV+cART+Ig | HIV+cART+CD24-Fc |
| 0.24 | 0.14 | 0.40 |
| 0.22 | 0.11 | 0.22 |
| 0.23 | 0.18 | 0.31 |
| 0.30 | 0.26 | 0.32 |
| 0.27 | 0.19 | 0.22 |
|  | 0.19 | 0.19 |

| Fig 2E | | |
| --- | --- | --- |
| mock | HIV+cART+Ig | HIV+cART+CD24-Fc |
| 0.560 | 0.750 | 1.170 |
| 1.140 | 0.323 | 4.210 |
| 5.470 | 0.450 | 3.100 |
| 0.820 | 0.380 | 5.340 |
| 0.800 | 0.360 | 1.140 |
|  | 0.430 | 0.590 |

| Fig 6B | | |
| --- | --- | --- |
| mock | HIV+cART+Ig | HIV+cART+CD24-Fc |
| 1.00 | 0.95 | 1.44 |
| 1.00 | 1.00 | 1.50 |
| 1.00 | 0.94 | 1.22 |
| 1.00 | 1.16 | 1.51 |
| 1.00 | 0.99 | 2.08 |
| 1.00 | 1.01 | 0.92 |
| 1.00 | 0.99 | 1.39 |
| 1.00 | 0.97 | 1.26 |
| 1.00 | 1.04 | 0.98 |
| 1.00 | 1.03 | 1.01 |

| Fig 6D | | |
| --- | --- | --- |
| mock | HIV+cART+Ig | HIV+cART+CD24-Fc |
| 1.00 | 1.02 | 1.49 |
| 1.00 | 0.99 | 1.19 |
| 1.00 | 1.05 | 1.38 |
| 1.00 | 0.99 | 1.78 |

| Fig 6G | | |
| --- | --- | --- |
| mock | HIV+cART+Ig | HIV+cART+CD24-Fc |
| 1.00 | 1.34 | 0.92 |
| 1.00 | 0.99 | 0.89 |
| 1.00 | 0.99 | 1.18 |
| 1.00 | 0.85 | 1.60 |
| 1.00 | 1.00 | 1.93 |
| 1.00 | 1.17 | 1.61 |
| 1.00 | 1.11 | 1.41 |
| 1.00 | 1.08 | 1.75 |
| 1.00 | 0.97 | 1.25 |
| 1.00 | 0.98 | 1.17 |
