# Supplementary material for: CD24-Fc resolves inflammation and enhances anti-HIV CD8 T cells with polyfunctionality during HIV-1 infection under cART
Source: PLoS Pathog. 2025 Aug 8;21(8):e1012826. doi: 10.1371/journal.ppat.1012826 (PMC12349878; doi:10.1371/journal.ppat.1012826)
Supplement: S1 Table — Information about humanized mice (Related to Figs 1–3). (DOCX) [file ppat.1012826.s007.docx]

**S1 Table. Information about humanized mice (Related to Figs 1-3)**

| **Mouse ID** | **Gender** | **Group** | **hCD45% in splenocytes** | **hCD3% in hCD45** | **IFN-γ in blood (pg/ml)** | **IP-10 in blood (pg/ml)** | **IL-10 in blood (pg/ml)** |
| --- | --- | --- | --- | --- | --- | --- | --- |
| 3757 | female | mock | 77.2 | 70.4 |  |  |  |
| 3758 | female | mock | 52.3 | 85 |  |  |  |
| 3825 | male | mock | 43.3 | 93.6 | 0.27 | 14.10 | 3.90 |
| 3826 | male | mock | 47.3 | 97.3 | 0.00 | 11.43 | 1.41 |
| 3827 | male | mock | 78.6 | 96.4 | 0.00 | 43.26 | 2.40 |
| 3740 | male | HIV+cART | 58.2 | 64.1 | 10.68 | 168.87 | 72.81 |
| 3751 | male | HIV+cART | 72.4 | 80.1 | 2.64 | 115.05 | 17.16 |
| 3753 | male | HIV+cART | 69.7 | 73.7 | 3.96 | 27.18 | 11.85 |
| 3754 | female | HIV+cART | 93.1 | 62.7 | 28.26 | 166.69 | 66.06 |
| 3755 | female | HIV+cART | 25.1 | 45.2 | 11.68 | 127.33 | 23.19 |
| 3756 | female | HIV+cART | 58.4 | 66.3 | 26.29 | 157.00 | 67.70 |
| 3742 | female | HIV+cART +CD24-Fc | 54.1 | 81.5 | 1.29 | 21.45 | 6.87 |
| 3744 | female | HIV+cART +CD24-Fc | 70 | 86.7 | 3.45 | 95.88 | 7.83 |
| 3745 | female | HIV+cART +CD24-Fc | 59.4 | 83.9 | 4.32 | 74.16 | 17.16 |
| 3748 | male | HIV+cART +CD24-Fc | 44.3 | 86.1 | 4.44 | 96.99 | 7.35 |
| 3750 | male | HIV+cART +CD24-Fc | 23.2 | 75.4 | 3.63 | 0.00 | 4.38 |
| 3752 | male | HIV+cART +CD24-Fc | 31.9 | 85.6 | 2.13 | 85.26 | 11.76 |
